# How do educational disparities in smoking develop during early life? A Swedish longitudinal study

**DOI:** 10.1016/j.ssmph.2021.100859

**Published:** 2021-06-30

**Authors:** Laura Wells, Viveca Östberg

**Affiliations:** Department of Public Health Sciences, Stockholm University, SE-106 91, Stockholm, Sweden

**Keywords:** Young adults, Education, School performance, Socioeconomic position, Smoking, Life course

## Abstract

Smoking contributes to health inequalities, but how social inequalities in smoking develop in early life remains unclear. This study examines how measures of education attained over the early life course (representing socioeconomic position of origin, socioeconomic position of destination, and in-between) contribute to smoking behavior in a Swedish longitudinal sample. We used data obtained from the Swedish Level-of-Living Surveys in addition to national register data. Young adults (aged 20–28, n = 749) self-reported their educational attainment and smoking behavior (initiation and cessation) in 2010. Ten years earlier, their parents self-reported their own education and smoking behavior. We used linked register data on school performance in adolescence (in grade 9). Logistic regression models showed that lower parental education, lower adolescent school performance, and low young adult educational attainment were respectively associated with young adult smoking initiation. The association between parental education and young adult smoking initiation was explained by adolescent school performance and not parental smoking. Young adult smoking cessation was associated with high parental education and high adolescent school performance (marks in the top quartile), but only school performance remained significant in the final model, which included all measures of education and parental smoking. Results suggest that school performance in adolescence (which connects adolescents’ socioeconomic position of origin with their destination) may play an important role in how educational disparities in smoking form over the life course.

## Highlights

• Parental education was associated with young adult smoking initiation and cessation.
• However, adolescent school performance was more important for smoking behavior.
• Young adults' educational attainment was also relevant for smoking initiation.
• Parental smoking did not explain educational differences in offspring smoking.
• School performance may play an important role in producing smoking inequalities.

## 1 Introduction

Cigarette smoking has declined in Sweden since the 1970s (Swedish Council for Information on Alcohol and Other Drugs, 2017). Today, about 15% of the Swedish adult population smokes, with about 8% smoking daily and about 7% smoking from time to time (Swedish Council for Information on Alcohol and Other Drugs, 2020a"). Sweden has the lowest proportion of daily smokers in the European Union (Swedish Council for Information on Alcohol and Other Drugs, 2020a). However, smoking is a risk factor for 28 diseases and remains a leading contributor to the disease burden in Sweden (Agardh, Boman, & Allebeck, 2015). Moreover, smoking-related health problems disproportionately affect disadvantaged socioeconomic groups (Hiscock, Bauld, Amos, Fidler, & Munafò, 2012; Laaksonen, Rahkonen, Karvonen, & Lahelma, 2005; Östergren, Martikainen, & Lundberg, 2018). Thus, efforts to improve public health and health equity (a goal of the Swedish government, see Lundberg, 2018) would benefit from an understanding of what puts people ‘at risk of the risks,’ that is, how socioeconomic position affects the risk of smoking and the development of smoking-related ill-health (Phelan, Link, & Tehranifar, 2010).

The literature depicts a complex, entwined relationship between socioeconomic position and smoking. A more disadvantaged position in the social structure tends to confer a greater risk of: smoking initiation, a higher rate of dependence, nonadherence to cessation treatment and a lower rate of cessation, and smoking-related mortality (Hiscock et al., 2012; Jarvis & Wardle, 1999; Swedish Public Health Agency, 2018). Tobacco control interventions have oftentimes failed to reduce these differences (Brown, Platt, & Amos, 2014), leaving persisting (and in some cases increasing) socioeconomic disparities in smoking behavior and smoking-related mortality (Eek et al., 2010; Swedish Public Health Agency, 2020; Östergren et al., 2018).

One argument for why there persist socioeconomic differences in smoking prevalence is the lack of focus on how smoking develops within a life course perspective (Viner et al., 2015). That is, studies addressing the relationship between socioeconomic position and smoking have often focused on adults and disparities in their current smoking or smoking cessation (Maralani, 2013). However, the prevalence of current and former adult smoking is a function of smoking initiation, as only those who begin smoking can continue or quit. Many studies therefore do not address the primary importance of smoking initiation, particularly as symptoms of nicotine withdrawal can develop shortly after initiation (DiFranza et al., 2011). Indeed, there is evidence that educational differences in smoking initiation better explain educational differences in adult smoking than educational differences in smoking cessation (Maralani, 2013). Efforts to reduce socioeconomic differences in smoking behavior (and in smoking-related health problems) would therefore benefit from a better understanding of how socioeconomic position can affect the risk of smoking initiation.

However, this is a task made challenging by the early age at which initiation occurs. Smoking is largely established in adolescence and young adulthood, with adolescence being the most sensitive period for initiation (Nilsson, 2005; Amos, Hastings, Angus, Bostock, & Fidler, 2009). Smoking typically begins before a young person has developed their own socioeconomic position. Indeed, smoking is largely established before the completion of one's education (Maralani, 2013). This makes it difficult to measure socioeconomic position when initiation occurs and complicates conceptualizing a causal effect of socioeconomic position on smoking initiation (Maralani, 2014).

A common strategy has been to focus on the adolescent's socioeconomic position of origin (e.g., parental education, social class, or income), and such studies do tend to find socioeconomic differences in adolescent smoking initiation, though the causal mechanisms behind these associations remain unclear (Hanson & Chen, 2007). Such associations may reflect the influence of parental smoking (Tyas & Pederson, 1998; Amos et al., 2009; Singh-Manoux & Marmot, 2005) or other mechanisms. Another strategy has been to focus on the adolescent's socioeconomic position of destination. For example, academic orientation (attendance of a theoretical versus non-theoretical upper secondary program) has been found to be more important for daily smoking at age 18 than parental education (Hagquist, 2007). However, as smoking typically begins before age 18, it is difficult to disentangle how achieved education (in upper secondary school or beyond) influences smoking initiation.

A third strategy is to focus on a measure of own education situated ‘in-between’ the individual's socioeconomic position of origin and their destination. School performance in adolescence is one such measure. School performance is a marker of the school's sorting of students into educational tracks, which affects the social stratification in adulthood (Elstad, 2010). Adolescent school performance is also linked to parental education and is an important part of how cultural capital is transferred inter-generationally (Andersen & Hansen, 2012). Moreover, school performance is closely tied to an adolescent's lived experience, which is likely to be important for smoking initiation. It has been argued that adolescents who receive low marks internalize their normative value (Elstad, 2010). Poor school performance could cause low self-esteem and anxiety about one's future success, for which smoking could be used as a coping mechanism (Elstad, 2010). Indeed, there is evidence that poor school performance is a risk factor for smoking initiation (Audrain-McGovern et al., 2004; Doku, Koivusilta, Rainio, & Rimpelä, 2010; Ellickson, Mcguigan, & Klein, 2001; Leatherdale, Hammond, & Ahmed, 2008) and that parental education explains only part of this association amongst Canadian adolescents (Morin, Rodriguez, Fallu, Maïano, & Janosz, 2012).

### 1.1 Aim of the study

In summary, understanding how smoking becomes socially differentiated is important from a public health perspective, but not enough of a focus has been placed on processes occurring in early life, when initiation occurs. This is in part because it is difficult to examine how socioeconomic disparities in smoking develop before education is achieved. The aim of this study is thus to disentangle how different aspects of education relevant to early life (i.e., measures of education representing socioeconomic position of origin, socioeconomic position of destination, and in-between) relate to smoking behavior. To this aim, we will examine how parental education, adolescent school performance, and young adult educational attainment associate with smoking initiation and cessation, measured in young adulthood at age 20–28 in a Swedish longitudinal sample. We will also assess the role of parental smoking to examine whether it may account for an association between parental education and young adult smoking behavior. As smoking initiation typically occurs before adulthood, but cessation is a behavior that can occur throughout the life course, this study will focus on initiation but will also examine whether aforementioned measures of education relate to early life smoking cessation.

## 2 Material and methods

### 2.1 Study population

This study uses longitudinal survey data from the 2000 and 2010 renditions of the Swedish Level-of-Living Survey (*Levnadsnivåundersökningen*, abbreviated as LNU), complemented with information from national registers. The LNU is a nationally representative study of 1/1000 of the Swedish adult population age 18–75 years (Bygren, Gähler, & Nermo, 2004). Structured interviews cover living conditions in a broad sense, including education and some health behaviors. In 2010, interviewers had access to some linked register data, i.e., young adults’ educational attainment is based on register data which was then confirmed or updated by the participant.

The study population is a cohort that first participated in the Child-LNU in 2000 as adolescents aged 10–18. They were recruited through a parent's participation in the LNU 2000. Of 1529 eligible adolescents, 1304 (85%) agreed to participate in the Child-LNU (for more information see Jonsson & Östberg, 2010). Ten years later, they were also invited to participate in the LNU 2010, now as young adults aged 20–28. Of the original 1304 adolescents who participated in the Child-LNU 2000, it was possible to follow up with 1290 in 2010, of which 809 (63%) agreed to participate in the LNU 2010. Participation was somewhat higher among women, in younger ages, if parents had a higher socioeconomic position, if parents were born in Sweden, and if the young adult had lived in a two-parent household in 2000. From this sample, any individuals who did not have matched register data on official school marks in grade 9 (n = 59) or were missing data on any of the study variables (n = 1) were excluded, leaving a final analytical sample of 749 young adults.

Parental information was drawn from the LNU 2000 and the Partner-LNU 2000, which was an abbreviated questionnaire completed by cohabitating partners of adult participants of the LNU 2000 (Jonsson & Östberg, 2010). All participants in the study provided informed consent. The Regional Ethics Committee of Stockholm (EPN) approved this study.

### 2.2 Variables

*Parental education* reflects the highest level of completed education in the household: (1) Tertiary degree (i.e., a college or university degree, which corresponds to 13+ years of education); (2) Upper secondary degree (corresponds to 11–12 years of education); (3) Compulsory degree or less education (9 or fewer years of education). Information from one parent was used when data was missing from the second parent (applicable for 11% of offspring who lived in two-parent households in 2000).

*Adolescent school performance* is measured as official school marks from the spring term of 9th grade (the final grade of compulsory school) when participants were 15–16 years old. School marks were obtained through register data, which was matched to survey participants by Statistics Sweden. Marks ranged from 0 to 320, which is the summation of 16 subjects, where each subject can be scored between 0 and 20. At this time, 20 points corresponded to a MVG, which means “excellent”, 15 points corresponded to a VG, which is “very good”, 10 points to an G, which is “good”, and 0 points to an IG, which is a failing grade (Swedish National Agency for Education, 2012). Marks were divided into quartiles.

Some young adults in our sample were too young to have graduated with a tertiary (college or university) degree, but all participants would have had the opportunity to graduate with an upper secondary degree. In Sweden, upper secondary education (*gymnasium)* is optional but highly attended. Today, and for this sample of young adults, upper secondary education consists of three years of study, typically conducted between the ages of 16–19. Most young adults (72% in 2009) receive final upper secondary marks by age 20 (Swedish National Agency for Education, 2010). We therefore chose to measure *young adult educational attainment* using three categories: (1) Upper secondary degree from a theoretical program or any post-upper secondary education; (2) Upper secondary degree from a vocational program; (3) Compulsory degree or less education. The distinction between theoretical and vocational upper secondary programs (sometimes called academic orientation) reflects different academic tracks: A higher proportion of students attending a theoretical program go on to pursue a tertiary (college or university) degree (Statistics Sweden, 2009). This measure has also been found to associate with Swedish young adult alcoholic drinking patterns (Wells & Östberg, 2018).

*Parental smoking* and *young adult smoking* were assessed using the question, ‘Do you smoke?’ Response options were ‘Yes, 10 cigarettes or more a day’; ‘Yes, less than 10 cigarettes a day’; ‘No, have given up’; ‘No, have never smoked.’ To assess young adult smoking behavior, we first examined smoking initiation by comparing ever smokers (combining the first three categories) with never smokers (the last category). We then made a separate comparison to assess smoking cessation by comparing former smokers (the third category) with current smokers (the first two categories), thereby removing never smokers (the last category) from the analysis. Parental smoking reflects one parent's smoking behavior as smoking was only reported in the LNU 2000 and not the Partner-LNU 2000. Two groups were created: current smokers (first two categories) and non-current smokers (last two categories).

*Age* in years, *gender* (male or female)*,* and *family composition* (living in a single or two-parent household in 2000) were included as covariates.

### 2.3 Data analysis

To examine how educational disparities in smoking develop during early life, we examined education as it is attained in a temporal order, i.e., first parental education, then adolescent school performance, and then young adult educational attainment. This allowed us to examine whether later measures of education (adolescent school performance or young adult educational attainment) might account for an association between earlier measures of education (parental education or adolescent school performance) and young adult smoking behavior (initiation or cessation). We also examined whether an association between parental education and young adult smoking behavior could be attributed to parental smoking.

Associations between study variables and young adult smoking behavior (initiation or cessation) were assessed through binary logistic regression. Average marginal effects (AME) were estimated as they are more reliable than odds ratios when comparing estimates across logistic regression models (Mood, 2010). AME can be interpreted as differences in predicted probabilities, i.e., they show the average change in the probability of smoking initiation (or cessation) with a one-unit increase in the predictor variable (Williams, 2016). As the sample includes individuals who lived in the same household in 2000 (siblings or stepsiblings), we estimated clustered robust standard errors in all analyses, with each individual household defined as one cluster. The full sample includes 575 unique households. Analysis was conducted using Stata 14.2.

## 3 Results

Descriptive statistics are shown in Table 1. Young adults in the sample were 48% male and 52% female with a mean age of 23. Most (71%) young adults reported having never smoked cigarettes, while 15% of the sample were current smokers and 14% were former smokers. Thus 29% were ever smokers (i.e., experienced smoking initiation). Within this group of ever smokers, 48% had already quit (i.e., experienced smoking cessation).

**Table 1:** Descriptive statistics by smoking status.

	All (n = 749)	Current smokers (n = 113)	Former smokers (n = 106)	Never smokers (n = 530)
	**n (%)**	**n (row %)**	**n (row %)**	**n (row %)**
**Parental education**				
Tertiary degree	195 (26.0)	19 (9.7)	31 (15.9)	145 (74.4)
Upper secondary degree	497 (66.4)	78 (15.7)	65 (13.1)	354 (71.2)
≤ Compulsory degree	57 (7.6)	16 (28.1)	10 (17.5)	31 (54.4)
				
**Parental smoking status**				
Non-smoker	597 (79.7)	75 (12.6)	80 (13.4)	442 (74.0)
Current smoker	152 (20.3)	38 (25.0)	26 (17.1)	88 (57.9)
				
**Adolescent (grade 9) school performance**				
1st (highest) quartile	183 (24.4)	7 (3.8)	16 (8.7)	160 (87.4)
2nd quartile	174 (23.2)	12 (6.9)	24 (13.8)	138 (79.3)
3rd quartile	202 (27.0)	38 (18.8)	35 (17.3)	129 (63.9)
4th (lowest) quartile	190 (25.4)	56 (29.5)	31 (16.3)	103 (54.2)
M±SD (range 0–320)	221 ± 56	184 ± 59	209 ± 53	231 ± 52
				
**Young adult educational attainment**				
≥ Theoretical upper secondary degree	370 (49.4)	31 (8.4)	45 (12.2)	294 (79.5)
Vocational upper secondary degree	320 (42.7)	64 (20.0)	52 (16.3)	204 (63.8)
≤ Compulsory degree	59 (7.9)	18 (30.5)	9 (15.3)	32 (54.2)
				
**Gender**				
Male	358 (47.8)	45 (12.6)	49 (13.7)	264 (73.7)
Female	391 (52.2)	68 (17.4)	57 (14.6)	266 (68.0)
				
**Family composition**				
Two-parent household	684 (91.3)	100 (14.6)	97 (14.2)	487 (71.2)
Single-parent household	65 (8.7)	13 (20.0)	9 (13.9)	43 (66.2)
				
**Age in years, M**±**SD**	23.1 ± 2.3	22.9 ± 2.1	23.5 ± 2.4	23.1 ± 2.3

*Note.* M = mean. SD = standard deviation.

Table 2 shows how measures of education in early life associate with smoking initiation. In crude analyses, parental education, adolescent school performance, and young adult educational attainment were respectively associated with smoking initiation. Compared to young adults whose parents held a tertiary degree, young adults whose parents had at most a compulsory degree had a 20% higher probability (*p* < 0.01) of having started smoking. Adolescent school performance was associated with smoking initiation in a graded pattern: Young adults with marks in the 2nd quartile (AME = 0.079, *p* < 0.05), 3rd quartile (AME = 0.250, *p* < 0.001), and even more so the 4th (lowest) quartile (AME = 0.356, *p* < 0.001) had a higher probability of smoking initiation than those with marks in the highest quartile. Young adults who held a vocational upper secondary degree (AME = 0.189, *p* < 0.001) and even more so a compulsory degree (AME = 0.314, *p* < 0.001) versus a theoretical upper secondary degree or higher education were more likely to have started smoking. When parental education and adolescent school performance were included in the same model, the association between parental education and smoking initiation was attenuated (Model 1). Young adult educational attainment and adolescent school performance were significantly associated with smoking initiation when all three measures of education were included in the same model (Model 2). Having a smoking parent was associated with a 14% higher probability (*p* < 0.001) of smoking initiation, but parental smoking did not substantially account for the association between parental education and smoking initiation (Model 3). In the full model, lower (bottom 50%) adolescent school performance, low young adult educational attainment, and parental smoking act as independent risk factors for smoking initiation (Model 4).

**Table 2:** Binary logistic regression: Average marginal effects (AME) and 95% confidence intervals (CI) for smoking initiation (being an ever vs. never smoker, n = 749).

	Crude		Model 1		Model 2		Model 3		Model 4	
	AME	95% CI	AME	95% CI	AME	95% CI	AME	95% CI	AME	95% CI
Parental education										
Tertiary (ref.)	0		0		0		0		0	
Upper secondary	0.033	-.042 .109	−0.022	-.098 .054	−0.036	-.114 .041	0.018	-.058 .094	−0.045	-.124 .033
≤ Compulsory	0.196**	.048 .345	0.047	-.081 .176	0.017	-.111 .145	0.177*	.023 .332	0.007	-.124 .139
										
**Adolescent (grade 9) school perf.**										
1st (highest) quartile (ref.)	0				0				0	
2nd quartile	0.079*	.003 .154	0.080*	.004 .157	0.079	-.002 .159			0.079	-.004 .161
3rd quartile	0.250***	.169 .332	0.251***	.168 .334	0.233***	.146 .320			0.227***	.138 .316
4th (lowest) quartile	0.356***	.271 .440	0.351***	.266 .436	0.300***	.204 .396			0.285***	.189 .381
										
**Young adult educational attainment**										
≥ Theoretical US (ref.)	0				0				0	
Vocational US	0.189***	.120 .259			0.081*	.002 .159			0.078*	.001 .156
≤ Compulsory	0.314***	.175 .453			0.203**	.065 .341			0.207**	.071 .344
										
**Parental smoking status**										
Non-smoker (ref.)	0						0		0	
Current smoker	0.142***	.067 .218					0.136***	.060 .213	0.098**	.025 .170

*Note.* US = Upper secondary. All analyses are adjusted for age, gender, and family composition. Crude analyses include one study variable at a time. Model 1 includes parental education and adolescent school performance. Model 2: Model 1 & young adult educational attainment. Model 3 includes parental education and parental smoking status. Model 4 includes all study variables. *p < 0.05*;* **p < 0.01*;* ***p < 0.001.

Table 3 shows how measures of education associate with smoking cessation in early life. In crude analyses, compared to young adults whose parents held a tertiary degree, young adults whose parents held at highest a compulsory degree had a 24% lower probability (*p* < 0.05) of having quit smoking. Adolescent school performance was also associated with smoking cessation in crude analysis: Young adults with marks in the lowest quartile had a 37% lower probability (*p* < 0.01) of having quit smoking than those with marks in the highest quartile. Model 1 shows how, when parental education and adolescent school performance were assessed simultaneously, the association between parental education and smoking cessation was no longer significant. As young adult educational attainment and parental smoking were not significantly associated with smoking cessation, adolescent school performance was the only significant factor associated with smoking cessation in the full model.

**Table 3:** Binary logistic regression: Average marginal effects (AME) and 95% confidence intervals (CI) for smoking cessation (being a former vs. current smoker, n = 219).

	Crude		Model 1		Model 2	
	AME	95% CI	AME	95% CI	AME	95% CI
Parental education						
Tertiary (ref.)	0		0		0	
Upper secondary	−0.139	-.306 .028	−0.110	-.280 .059	−0.104	-.281 .073
≤ Compulsory	−0.242*	-.477;-.008	−0.127	-.375 .122	−0.116	-.377 .146
						
**Adolescent (grade 9) school perf.**						
1st (highest) quartile (ref.)	0		0		0	
2nd quartile	−0.055	-.304 .193	−0.047	-.299 .205	−0.048	-.302 .206
3rd quartile	−0.209	-.425 .008	−0.195	-.414 .025	−0.190	-.411 .031
4th (lowest) quartile	−0.366**	-.579;-.154	−0.343**	-.561;-.125	−0.329**	-.558 .101
						
**Young adult educational attainment**						
≥ Theoretical US (ref.)	0				0	
Vocational US	−0.132	-.277 .013			−0.009	-.159 .141
≤ Compulsory	−0.211	-.442 .019			−0.058	-.296 .181
						
**Parental smoking status**						
Non-smoker (ref.)	0				0	
Current smoker	−0.086	-.234 .062			−0.026	-.175 .122

*Note.* US = Upper secondary. All analyses are adjusted for age, gender, and family composition. Crude analyses include one study variable at a time. Model 1 includes parental education and adolescent school performance. Model 2 includes all study variables. *p < 0.05*;* **p < 0.01*;* ***p < 0.001.

## 4 Discussion

The aim of this study was to disentangle how different aspects of education relate to smoking behavior in early life in a Swedish longitudinal sample. We found that parental education, adolescent school performance, and young adult educational attainment were respectively associated with young adult smoking initiation, but that the association between parental education (a dimension of socioeconomic position of origin) and smoking initiation was explained by adolescent school performance. We also found an association between young adult educational attainment (a marker of socioeconomic position of destination) and young adult smoking initiation that was independent of parental education and adolescent school performance, indicating that low achieved education (or an expectation of low achieved education) is relevant for smoking initiation. Lastly, parental education and adolescent school performance were respectively associated with young adult smoking cessation, but only adolescent school performance was significantly associated with smoking cessation when both parental education and school performance were examined simultaneously. These findings are discussed below.

### 4.1 Adolescent school performance was more important per se for smoking initiation than parental education

Adolescent school performance was inversely patterned with smoking initiation, i.e., the probability of smoking initiation increased with lower school performance. Thus, all grade 9 students with marks outside the top 25% had a higher risk of having experienced smoking initiation by young adulthood. Indeed, adolescent school performance accounted for the association between parental education and smoking initiation, which suggests that school performance may serve as an important link between socioeconomic position of origin and smoking initiation.

Adolescence may be the most important life stage for understanding how smoking becomes socially differentiated (Due et al., 2011; Maralani, 2013; Viner et al., 2012). As adolescents spend a substantial amount of time in school, one would expect aspects of the school environment and school experience to influence smoking initiation. Indeed, there is evidence for school-contextual effects on smoking (though more and less advantaged schools in Sweden have been reported as having a higher percentage of smoking students, see Olsson and Modin, 2020, C.A.N., 2020b). The school is also a powerful social institution which grades, ranks, and sorts students into hierarchies of learning capacity and academic ability, and thus conveys the “relative worth of individuals,” by “tell [ing] the adolescent whether they are headed towards prestigious, well-paid occupational positions or towards low-paid, low-status work” (Elstad, 2010, p. 141). This formative experience is thought to shape adolescent identities and health behaviors. For instance, adolescents who are not top performers may take up smoking because of the stress and anxiety that results from being found less worthy, or because they become more interested in assuming a countercultural social identity, which smoking may facilitate. Adolescents who receive low marks may also find their future to be less certain, which may cause them to become less concerned with protecting their future health or avoiding risks. Poor performing adolescents may also anticipate their future social class and see smoking as a way of fitting into future class environments (Elstad, 2010). While testing such mechanisms was outside the scope of this study, it is well-known that stress and anxiety motivate smoking (Dijk, de Nooijer, Heinrich, & de Vries, 2007), and there is evidence that future discounting and risk aversion are associated with smoking behavior (Daly, Delaney, & Baumeister, 2015; Goto, Takahashi, Nishimura, & Ida, 2009).

However, other factors may also be relevant to an association between school performance and smoking initiation; for example, an association between adolescent school performance and smoking initiation may be explained by poor mental health, low self-esteem, adverse childhood experiences, or developmental disorders like Attention Deficit/Hyperactivity Disorder, which could negatively impact the adolescent's school performance and make smoking initiation more likely (Joffer et al., 2014; Russell, Ford, Williams, & Russell, 2016; Van Loon, Tijhuis, Surtees, & Ormel, 2005). More research is needed examining how adolescent school performance may influence smoking initiation, particularly as schools are important actors in efforts to equalize early life social inequalities.

### 4.2 Parental smoking did not explain educational differences in smoking initiation

Socioeconomic position of origin is often thought to influence adolescent smoking initiation through parental smoking (Hanson & Chen, 2007). That is, parents can contribute to the reproduction of socioeconomic disparities in smoking in the next generation if they model their own socially differentiated smoking behavior to their children (Singh-Manoux & Marmot, 2005). While we did find that parental smoking was associated with parental education (results not shown), parental smoking did not substantially explain differences in smoking initiation by parental education. This may indicate that there are other factors connecting parental education with smoking initiation, and in this study, we did find that adolescent school performance accounted for the association between parental education and smoking initiation. However, our results may have differed if we had access to both parents’ smoking behavior or if we had examined parental social class or income instead of parental education.

### 4.3 Young adult educational attainment was relevant for smoking initiation

This study found that a marker of future socioeconomic position (young adult educational attainment) was associated with smoking initiation. Indeed, young adult educational attainment was associated with smoking initiation independent of parental education and adolescent school performance. It is difficult to disentangle what this association represents, but some insight may be found in how we measured educational attainment.

In this study, educational attainment reflects both vertical and horizontal dimensions of education, i.e., both the level of completed education and the type of upper secondary program. Regarding the vertical dimension, our results suggest that young adults who only obtained a compulsory degree were especially vulnerable to start smoking. It is possible that smoking began, however, when it was only *anticipated* that the young person would not attend or graduate from upper secondary school. Another explanation is that there may be shared risk factors for smoking initiation and low educational attainment (e.g., traumatic events, developmental disorders, mental health problems).

Regarding the horizontal dimension, previous studies have shown that enrollment in different types of upper secondary programs is associated with smoking behavior (Hagquist, 2007; Robert et al., 2019). Moreover, in a study of grade 9 Swedish students, those whom had applied but not yet enrolled in a non-theoretical upper secondary program were more likely to have started smoking (Hagquist, 2000). Thus, before entering different upper secondary educational environments (which are catered to different occupations and may have different smoking norms), there are differences in adolescents’ smoking behavior by future academic orientation. This could occur if educational aspirations are associated with smoking behavior (e.g., see Brook, Balka, Zhang, Pahl, & Brook, 2011) and may be consistent with a social causation framework if aspirations are influenced by socioeconomic position of origin (e.g., see Geckova, Tavel, van Dijk, Abel, & Reijneveld, 2010).

### 4.4 Adolescent school performance was also associated with smoking cessation

This study found that parental education and adolescent school performance were respectively associated with smoking cessation, though only adolescent school performance was significant when both were estimated in the same model. No other factors were significantly associated with smoking cessation, which could reflect an insufficient power to detect significant differences amongst a small subsample of ever smokers.

Few studies have examined whether adolescent school performance is associated with *both* smoking initiation and cessation. However, our results are in line with a study in the United States which found that self-reported school performance at age 13 was associated with initiation and cessation at age 18 (Ellickson et al., 2001). That school performance was associated with both initiation and cessation may indicate that school performance (which is itself associated with socioeconomic position of origin and socioeconomic position of destination) plays an important role in how smoking becomes socially differentiated over the life course. Indeed, our results suggest that adolescents who receive low marks may be less motivated to quit or may find it more difficult to quit than those who receive high marks. It is possible that low marks induce school-performance related stress, which could make quitting less likely if smoking is used as a coping mechanism for dealing with stress. More research should examine how adolescents cope with poor school performance, particularly as interviews with Swedish adolescents suggest that many see school performance-related stress as a problem (Låftman, Almquist, & Östberg, 2013).

### 4.5 Strengths and limitations

This study was aided by the use of longitudinal survey data, which allowed us to examine inter-generational and intra-generational educational differences in smoking behavior. Another strength was the use of national register data within the surveys, which enabled us to obtain an accurate and up-to-date measure of young adult educational attainment. We were also able to ascertain adolescent school performance through register data and not through self-report, as is more common in the literature. However, our measure of parental smoking only reflected one parent's smoking behavior, which may have underestimated the effect of parental smoking. It was also not possible to determine at what age smoking initiation or cessation occurred. Use of longitudinal survey data also constrained the sample size and limited our ability to look at more refined age groups, regional differences (which may relate to the availability of cigarettes and to different norms, Hagquist, Sundh, & Eriksson, 2007), or stratify analyses by gender (which may be important for understanding smoking behavior in Sweden, Hagquist, 2007; Nilsson, 2005).

Furthermore, the use of multiple regression analysis did not allow for the estimation of all indirect effects in hypothesized models (e.g., how parental education associates with young adult educational attainment, directly and via adolescent school performance). Social selection to higher education is well-established in Swedish literature (see, e.g., Erikson & Rudolphi, 2010); however, future studies could employ structural equation modeling to better examine how educational pathways connect parental education with young adult smoking behavior.

It was not possible to examine use of snus (smokeless snuff tobacco), which in Sweden is quite common and has been linked to cigarette smoking cessation (Ramström & Foulds, 2006). Data on peer smoking was also unavailable. However, friendship group selection is itself associated with other factors (like school performance, see Robert et al., 2019), so peer influence is not alone a sufficient explanation for understanding how smoking becomes socially differentiated (Arnett, 2007; Elstad, 2010). We also did not examine cognitive ability; however, a recent study employing Mendelian randomization did not find that general cognitive ability explained educational differences in smoking initiation or cessation (Sanderson, Smith, Bowden, & Munafò, 2019).

### 4.6 Conclusions

There have been recent calls to make Sweden smoke free (Hallengren, 2017) and to reduce health inequalities (Lundberg, 2018), respectively. However, policy efforts to lower the overall smoking prevalence have not always reduced relative social inequalities in smoking behavior and relative social inequalities in health (Brown et al., 2014; Frohlich & Potvin, 2008). Achieving greater equity in health would benefit from efforts to prevent smoking initiation among disadvantaged groups (Maralani, 2013). Our study contributes to this ambition by providing insight into how educational disparities in smoking form during early life. In particular, our results suggest that adolescent school performance may be relevant to early life smoking initiation and cessation. More research is needed, but a policy entry point could be in providing academic and social support to adolescents with low school performance. It is difficult to recruit socially disadvantaged adolescents to smoking prevention programs (e.g., Hedman et al., 2015). Moreover, adolescents are well aware of the health risks of smoking (Rosendahl, Galanti, Gilljam, & Ahlbom, 2005), so efforts to prevent initiation among targeted groups should not assume that smoking is motivated by less knowledge or lower cognitive ability. As smoking begins early in life, and is associated with socioeconomic position of origin, there is an ethical responsibility to frame smoking as socially structured and to improve the health of young people born into families with less structural advantages.

## Ethical statement

We declare no conflicts of interest. This article is a part of a study that has been approved by the Regional Ethics Committee of Stockholm (EPN, #2016/252–31/5).

## Author statement

**Laura Wells:** Conceptualization; Methodology; Formal analysis; Writing - Original Draft; Writing - Review & Editing; Visualization.

**Viveca Östberg:** Conceptualization; Methodology; Writing - Review & Editing; Funding acquisition.

## Funding

This study was financially supported by the Swedish Research Council for Health, Working Life and Welfare (Grant No. 2013–0159; 2015–00399).
